# Do Only Small Uremic Toxins, Chromophores, Contribute to the Online Dialysis Dose Monitoring by UV Absorbance?

**DOI:** 10.3390/toxins4100849

**Published:** 2012-10-18

**Authors:** Jürgen Arund, Risto Tanner, Fredrik Uhlin, Ivo Fridolin

**Affiliations:** 1 Department of Biomedical Engineering, Technomedicum, Tallinn University of Technology, Ehitajate tee 5, 19086 Tallinn, Estonia; Email: risto.tanner@kbfi.ee (R.T.); fredrik.uhlin@lio.se (F.U.); ivo@cb.ttu.ee (I.F.); 2 Laboratory of Chemical Physics, National Institute of Chemical Physics and Biophysics, Akadeemia tee 23, 12618 Tallinn, Estonia; 3 Department of Nephrology UHL, County Council of Östergötland, Department of Medical Health Sciences, Faculty of Health Sciences, Linköping University, Linköping, Sweden

**Keywords:** uremic toxins, hemodialysis, chromophores, retention solutes, absorption, ultraviolet-radiation, liquid-chromatography, dialysis dose, monitoring, spent dialysate

## Abstract

The aim of this work was to evaluate the contributions of the main chromophores to the total UV absorbance of the spent dialysate and to assess removal dynamics of these solutes during optical on-line dialysis dose monitoring. High performance chromatography was used to separate and quantify UV-absorbing solutes in the spent dialysate sampled at the start and at the end of dialysis sessions. Chromatograms were monitored at 210, 254 and 280 nm routinely and full absorption spectra were registered between 200 and 400 nm. Nearly 95% of UV absorbance originates from solutes with high removal ratio, such as uric acid. The contributions of different solute groups vary at different wavelengths and there are dynamical changes in contributions during the single dialysis session. However, large standard deviation of the average contribution values within a series of sessions indicates remarkable differences between individual treatments. A noteworthy contribution of Paracetamol and its metabolites to the total UV absorbance was determined at all three wavelengths. Contribution of slowly dialyzed uremic solutes, such as indoxyl sulfate, was negligible.

## 1. Introduction

The search for an easy and robust method for online tracking of a prescribed dialysis dose when dialysis is used as a treatment for patients with kidney failure is a long-term pursuit. Blood samples have been the main source of information concerning the efficiency of dialysis treatment during the history of search for a suitable parameter for dialysis dose description. The Kt/V value based on urea analyses in blood samples has been commonly accepted for the description of a delivered dialysis dose today. However, the method is error-prone in practice [1] and time-consuming, considering the time needed from blood draw until achieving the results. Urea itself does not exhibit toxic properties in concentrations found in the dialysis patients [2], and is not representative for removal of many uremic toxins regarded as groups of protein bound and middle molecules [3].

A principle for a non-invasive dialysis adequacy monitoring method was proposed by Gal *et al*. [4] proposing measuring UV absorbance in the spent dialysate at 254 nm. This method was not widely adopted at the time. A decade later, the principle of conductivity based dialysis monitoring was introduced utilizing the conductivity signal to assess the dialysis dose parameter Kt/V [5,6]. However, the precision of conductivity based Kt/V assessment appeared to be dependent on accurate estimation of total body water [7] and, therefore, not an ideal method for routine use. Also, online urea content monitoring in the spent dialysate has been used (Biostat 1000 Urea Monitor [8,9], Biotrack [10]). The equipment, which is rather cumbersome to handle and involves significant running costs, has not found wider acceptance. The observed relation between the online UV absorbance signal and the parameter Kt/V however, led a step closer to a robust, cheap and reliable way of dialysis monitoring [11]. Use of light emitting diodes made it possible to miniaturize the sensor and minimize the cost of the monitor, without any need for consumables [12].

Earlier studies have shown that UV absorptions at 280, 285 and 297 nm have a close correlation with urea-based dialysis dose estimation [11,13,14]. This made it possible to develop a clinically validated online dialysis adequacy monitoring system [15]. The system measures UV-absorption *versus* time in the spent dialysate at 280 nm and calculates Kt/V.

Because the UV method detects a range of solutes, it is sensitive to changes in chromophores’ content and the appearance ratio of different UV-absorbing molecules in the spent dialysate. Removal dynamics of different chromophores and contributions to the total UV absorbance are still unknown [16]. 

The aim of this study was to evaluate the contributions of the main chromophores to the total UV absorbance in the spent dialysate and removal dynamics during optical online dialysis dose monitoring.

## 2. Results

**Figure 1:**
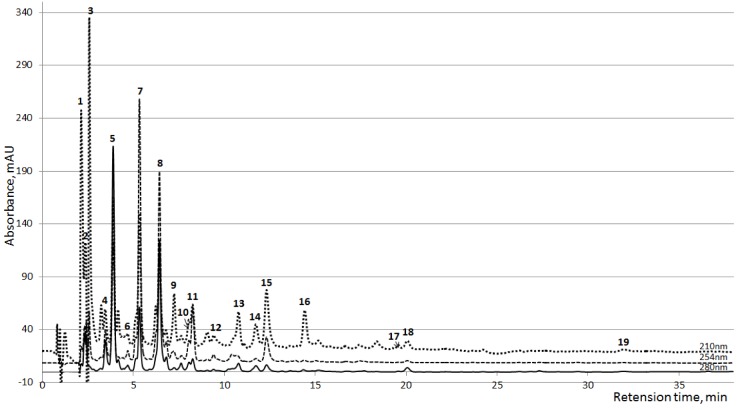
Averaged HPLC chromatograms of the spent dialysate collected 10 min after the start of the dialysis (*n*=24) at three wavelengths. 1,2: Unknown; 3: Creatinine; 4: Unknown; 5: Uric acid; 6: Hypoxanthine; 7: PAR Glucoronide; 8–10: Unknown; 11: PAR Sulfate; 12: Paracetamol (PAR); 13: Tryptophan; 14: Indoxyl Sulfate; 15: Hippuric acid; 16–18: Unknown; 19: Indole-3-acetic acid.

Thirty clearly resolved peaks of UV absorbing compounds were detected during the HPLC analysis. Seventeen of all peaks had major importance in some samples or prevalent importance in all samples (Figure 1), from these, 10 were identified on the basis of comparisons of the MS-spectra, UV-spectra and the retention time with the corresponding reference substances (Table 1). Identified peaks were grouped considering the widely accepted classification of uremic retention solutes [17]. An additional three peaks (7, 11 and 12, Figure 1), identified as Paracetamol (PAR; *N*-Acetyl-*p*-Aminophenol) and metabolites PAR glucuronide and PAR sulfate, were found from the samples of 33 dialysis sessions out of 48 (including both HD and HDF sessions).

Also, five prevalent but unidentified chromatographic peaks were grouped together (Table 1). The group of “All Other Solutes” (AOS) consists of the peaks that had no prevalent signal in the chromatograms or were not clearly identified as separate peaks. This group involves an unknown number of solutes, which separately had very low UV signal, but summed together had noticeable importance in total UV absorbance. 

**Figure 2:**
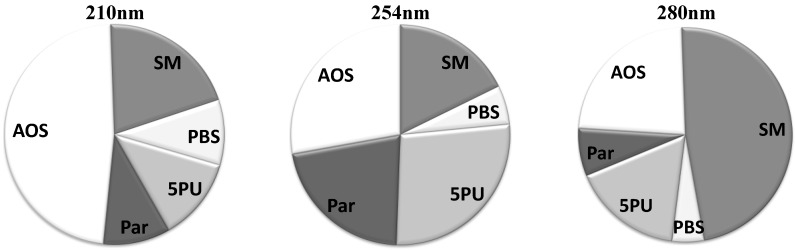
Average contribution of groups of chromatographic peaks to the total UV absorbance in the spent dialysate, including start, end and tank collection samples.**SM**: Small molecules; **PBS**: Protein-Bound Solutes; **5PU**: 5 Prevalent Unidentified Peaks; **Par**: Paracetamol and its metabolites; **AOS**: All other Solutes.

The average relative contributions for all five solute groups at three different wavelengths are illustrated in Figure 2. The group of “Small Molecules” has prevalent contribution at 280 nm. However, at lower wavelengths this group loses dominance in the UV absorbance signal. At 254 nm the group of “5 Prevalent Unidentified Peaks (5PU)” has a significant role in the UV signal, but less significant at 210 and 280 nm. The UV absorbance signal is most complex at 210 nm, where nearly half of the signal consists of many solutes with low and very low UV absorbance at higher wavelengths.

**Figure 3:**
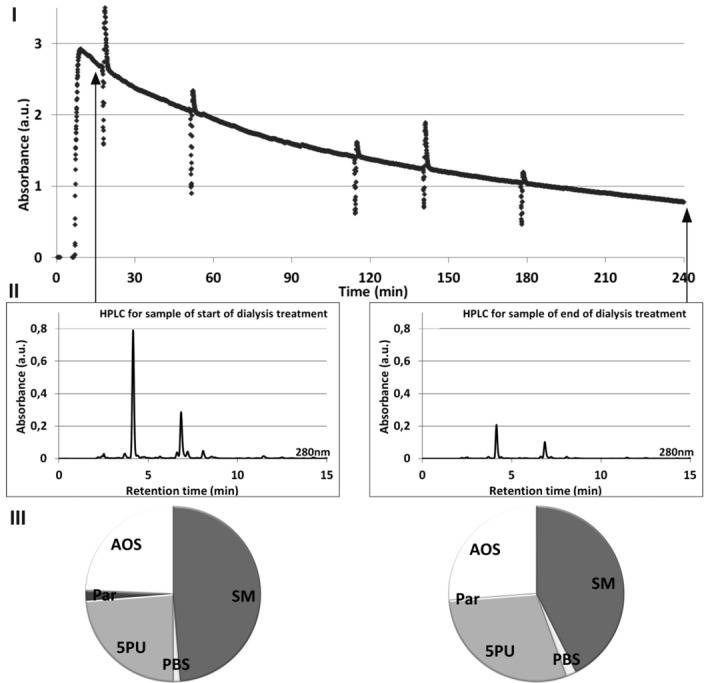
Relationship between online UV absorbance (**I**), HPLC signal (**II**) and the relative contribution of the HPLC peaks to the total UV absorbance at 280 nm (**III**) for a start and end of dialysate samples from a single dialysis treatment.

Figure 3 illustrates the relationship between online UV absorbance of spent dialysate monitored during dialysis session (**I**), HPLC signal (**II**), and the relative contribution of the HPLC peaks to the total UV absorbance (**III**), all signals acquired at 280 nm for a single dialysis treatment. The slope of online UV absorbance signal from the spent dialysate against time enables one to estimate the value of Kt/V (Figure 3I). Method for acquiring the online UV absorbance signal is described in detail elsewhere [18]. Self-tests of the dialysis machine occur as spikes in the signal. Difference in the height of the peaks on the Start and End chromatograms (Figure 3II) demonstrates a concentration decrease in uremic solutes during the dialysis.

**Table 1:** Grouping of solutes.

Grouping	Compound	Peak nr	RT, min	MW	Class
Small Molecules (**SM**)	Creatinine (Cr) *	3	2.5	113	Guanidines
	Uric acid (UA) *	5	4.0	168	Purines
	Hypoxanthine *	6	4.4	136	Purines
Protein-Bound Solutes (**PBS**)	Tryptophan (Trp)	13	11.4	204	Indoles
	Indoxyl Sulfate (IS) *	14	12.4	251	Indoles
	Hippuric acid (HA) *	15	13.0	179	Hippurates
	Indole-3-acetic acid (I3AA) *	19	31.8	175	Indoles
5 Prevalent Unidentified Peaks (**5PU**)	Unknown	1	2.4		
	Unknown	4	3.6		
	Unknown	8	6.8		
	Unknown	10	8.4		
	Unknown	18	20.7		
Paracetamol and its metabolites (**Par**)	Paracetamol Glucoronide	7	5.5	327	Glucuronides
	Paracetamol Sulfate	11	8.7	231	
	Paracetamol (PAR)	12	11.1	151	Acetanilides
All Other Solutes (**AOS**)	Unknown	2	2.3		
	Unknown	9	7.2		
	Unknown	16	14.4		
	Unknown	17	19.5		

* Note: Grouping according to EUTox (European Uremic Toxin Work Group) classification [17]; RT: chromatographic retention time (minutes), MW: molecular weight (g/mol).

The average relative contributions for all treatments to the total UV absorbance (Mean ± SD) in percentage for each peak and each solute group are given in Table 2 for start and end samples at three wavelengths. Characteristic dynamics of the contributions from the solutes and solute groups to the total UV absorbance can be distinguished by comparing the data from the start and the end of the dialysis session. Two peaks, identified as Hypoxanthine and I3AA, had very low UV signal value in the chromatograms, and were included into the group of AOS.

As seen from Table 2, the small molecule, Uric Acid (UA) is the main UV absorbing solute in the spent dialysate at 280 nm. During the dialysis, the importance of UA in the UV signal decreases significantly (*p* < 0.05). The decrease of UA contribution is concurrent to the increased contributions from other solute groups, being significant for groups “Protein-Bound Solutes” (PBS) and AOS (*p* < 0.05). As UA is a very important UV absorber in the spent dialysate, the high standard deviation value of UA contribution should be stressed. This indicates high variations between different patients and dialysis sessions (min *RC*_UA_ = 29%; max *RC*_UA_ = 75%, RC: relative contribution).

Solute contributions at 254 nm have the highest SD value. The main contribution comes from the group of 5 PU, where the peak Unknown 8 has the highest contribution to the UV signal.

At 210 nm, two solutes (UA and Creatinine) in the “Small Molecules” group are of major importance in the UV signal. Their contributions changed during the dialysis considerably (*p* < 0.05). Changes in the contribution inside the group of PBS were significant. However, no substantial difference in the contribution occurred for the start and end samples for the whole PBS group.

**Table 2:** Average contributions in percent for each peak and molecule group with a statistical comparison of the start and end samples of the spent dialysate at three wavelengths *).

	210 nm		254 nm		280 nm	
	Start	End	Start	End	Start	End
**Small molecules**	24.81 ± 8.02*	16.36 ± 4.88	18.63 ± 8.86	16.94 ± 7.44	50.07 ± 10.54 *	44.88 ± 9.73
Uric acid	10.30 ± 3.67*	6.80 ± 2.21	14.83 ± 7.51	12.98 ± 6.04	50.07 ± 10.54 *	44.88 ± 9.73
Creatinine	14.51 ± 5.37*	9.56 ± 4.77	3.80 ± 1.69	3.96 ± 1.62	0.00	0.00
**Protein-Bound Solutes**	9.75 ± 3.36 *	9.32 ± 2.61	6.04 ± 3.17	5.42 ± 2.72	3.82 ± 0.97 *	5.87 ± 1.59
Indoxyl Sulfate	1.89 ± 0.79*	2.57 ± 1.04	0.44 ± 0.38	0.33 ± 0.46	1.42 ± 0.50 *	2.44 ± 1.13
Tryptophan	1.68 ± 0.59 *	2.71 ± 0.73	0.51 ± 0.26	0.52 ± 0.64	1.21 ± 0.29 *	2.41 ± 0.69
Hippuric acid	6.18 ± 3.27 *	4.04 ± 2.18	5.09 ± 2.87	4.57 ± 2.76	1.19 ± 0.62	1.02 ± 0.58
**5 Prevalent Unidentified peaks**	12.72 ± 4.41	11.55 ± 3.73	26.88 ± 13.50	27.68 ± 12.52	15.80 ± 6.01	17.75 ± 5.18
Unknown1	0.00	0.00	0.00	0.00	0.93 ± 1.71	0.79 ± 1.32
Unknown4	2.55 ± 0.76 *	2.26 ± 0.83	4.21 ± 1.80	4.49 ± 1.81	2.86 ± 0.53 *	3.11 ± 0.48
Unknown8	6.49 ± 4.19	5.66 ± 3.35	18.76 ± 13.61	19.79 ± 13.15	9.16 ± 6.40	10.03 ± 5.86
Unknown10	2.86 ± 1.14 *	2.10 ± 0.90	3.27 ± 1.26 *	2.46 ± 1.38	1.75 ± 0.97 *	1.27 ± 1.10
Unknown18	0.82 ± 0.82	1.53 ± 0.97	0.64 ± 1.39	0.94 ± 1.54	1.10 ± 0.97 *	2.55 ± 1.72
**Paracetamol and metabolites**	10.01 ± 10.55	8.94 ± 8.80	21.49 ± 22.02	21.18 ± 21.31	7.37 ± 8.16	6.87 ± 7.40
Paracetamol	0.66 ± 0.51	1.08 ± 1.05	0.79 ± 0.78 *	1.40 ± 2.14	0.50 ± 0.52	0.73 ± 1.30
Paracetamol Glucoronide	7.26 ± 8.48	6.23 ± 6.81	16.62 ± 17.59	15.49 ± 15.89	5.59 ± 6.82	5.01 ± 5.73
Paracetamol Sulfate	2.09 ± 2.01	1.63 ± 1.55	4.08 ± 4.29	4.29 ± 4.39	1.28 ± 1.04	1.13 ± 1.13
**All Other Solutes**	42.72 ± 8.43 *	53.84 ± 6.79	26.97 ± 7.88	28.80 ± 7.93	22.95 ± 3.65 *	24.64 ± 3.71

* Note: Start values with asterisk indicate significant statistical differences (*p* < 0.05) between the values for start and end spent dialysate samples.

An alternative grouping of solutes was done on the basis of solutes’ Removal Ratios (RR) during the dialysis (Table 3). RR values for all the detected chromatographic peaks were calculated, and these values were compared with RRs of other chromatographic peaks using Student’s *t*-test. Chromatographic peaks the RRs of which were not significantly different were grouped together. Four groups with statistically different RR values were created. Creatinine, which had statistically different RR from both “High RR 1” and “High RR 2” groups, was placed under the latter group in Table 3 as the average RR value was closer to this group. Group of AOS were included to the group “High RR 2” as the RR values were statistically indifferent. The RR value of group of AOS corresponds to the summated change of peaks for the whole group, since it was impossible to evaluate the RR values for single peaks due to low concentration and/or insufficient separation on “end” chromatograms.

**Table 3:** Grouping of the solutes according to the removal ratio (RR), mean ± SD (%).

High RR 1		High RR 2		Low RR		Unstable RR	
UA	69.0 ± 11.2	Creatinine	63.1 ± 10.3	IS	48.1 ± 13.2	Trp	32.7 ± 23.0
HA	68.4 ± 10.4	Unknown 1	62.1 ± 9.0			PAR.	14.4 ± 64.4
Unknown10	72.7 ± 9.6	Unknown 4	62.3 ± 10.3			Unknown 18	−131.0 ± 309.1
PAR.Gluc	71.9 ± 15.0	Unknown 8	60.9 ± 10.5				
PAR.Sulf	64.4 ± 24.0	All other molecules	63.9 ± 11.1				

As Table 3 shows, the most indicative marker of low RR compounds in terms of UV-monitoring of the dialysis appears to be indoxyl sulfate without any severe rivalry by other common UV-chromophores in the spent dialysate.

**Figure 4:**
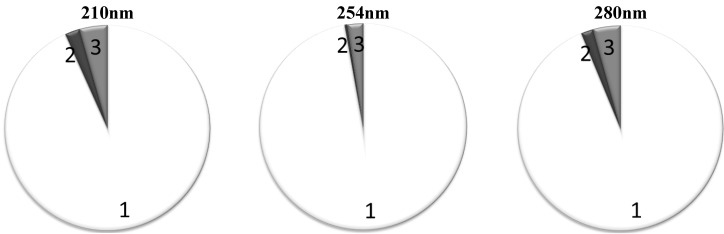
Average contribution to the total UV absorbance in percentage from the chromophores belonging to different RR groups, the criterion for the “High RR sum” group inclusion was average RR > 60%: 1: Solutes with high RR; 2: Solutes with low RR; 3: Solutes with unstable RR.

Recalculated average relative contributions (Mean ± SD) for alternative grouping based on RR values are given in Table 4. The groups with the highest RR, “High RR 1” and “High RR 2”, play a major role both at 280 and 254 nm. Also, they remain prevalent contributors at 210 nm. Both at 280 nm and 254 nm, the “High RR 1” and “High RR 2” groups together are responsible for around 95% of the total UV absorbance (Figure 4). The contribution of the IS as a marker of retention solutes with low RR remains inconsiderable at all wavelengths tested.

Figure 4 shows the average contribution to the total UV absorbance from the chromophores belonging into different RR based groups when the “High RR 1” and “High RR 2” groups were put together.

**Table 4:** Average contributions in percent of RR based groups of chromophores to the total UV absorbance in the spent dialysate (Mean ± SD).

		High RR 1	High RR 2	Low RR	Unstable RR
210 nm	Start	28.69 ± 11.28 *	66.26 ± 10.50 *	1.89 ± 0.79 *	3.16 ± 1.23 *
	End	20.79 ± 8.24	71.31 ± 8.24	2.57 ± 1.04	5.32 ± 1.31
254 nm	Start	43.89 ± 18.68	53.74 ± 18.01	0.44 ± 0.38	1.94 ± 1.50 *
	End	39.79 ± 15.84	57.03 ± 16.85	0.33 ± 0.46	2.85 ± 2.46
280 nm	Start	59.88 ± 6.78 *	35.90 ± 7.28	1.42 ± 0.50 *	2.81 ± 1.10 *
	End	53.30 ± 6.23	38.57 ± 6.55	2.44 ± 1.13	5.68 ± 2.01

* Note: Start values with asterisk indicate significant statistical difference (*p* < 0.05) between the values of start and end spent dialysate samples.

## 3. Discussion

This study adds an exciting supplement to the current knowledge about the removal dynamics of different chromophores and contributions to the total UV absorbance. The results indicate that: (i) a predominant part (>95%) of the UV absorbance signal in the spent dialysate originates from easily dialyzed uremic solutes with a high removal ratio, like uric acid; (ii) a noteworthy role of Paracetamol and its metabolites in the UV absorbance signal was determined at all three wavelengths; (iii) the contribution of UA changes during the dialysis treatment due to more efficient removal of small water soluble solutes, resulting in an increased contribution in other molecules; (iv) UV absorbance cannot be utilized to monitor the removal of slowly dialyzed uremic solutes (e.g., indoxyl sulfate); (v) an alternative grouping for uremic solutes based on removal ratios is proposed; (vi) a significant part of UV absorbance is caused by unidentified molecules.

Earlier research by Schoots [19] has found that removal ratios for known uremic toxins vary for each solute due to changes in protein binding during the dialysis treatment for protein-bound solutes and changes in clearances for different solutes. It can be concluded from the results presented in this work that a predominant part, roughly 95% of UV-absorbing uremic solutes in the range of absorbance between 254 and 280 nm, evidently do not belong to the group of firmly protein bound substances. The result does not support the earlier conclusion [4] that the UV absorbance at 254 nm should enable one to follow the elimination of accumulated plasma components with particular emphasis on slowly diffusible organic compounds of known or assumed toxicity. This study and several other earlier studies [19,20,21] have indicated that a major part of the UV signal originates from the small toxic solute of UA, which enables online monitoring of UA [22]. The small water soluble molecule, UA, has the most important role at the wavelength 280 nm where it is responsible for a major part of the total UV absorbance: the mean contribution of UA was about half (48%). The average removal ratio of the UA 69% is comparable to that of a traditional marker urea 71% in this study (unpublished result). These observations strongly empower the spreading practice of using online UV-monitoring to evaluate the dialysis process and to calculate a dialysis dose (KtV value) [23]. Furthermore, recent unpublished results from our research group show very good correlation between the UA concentration and the UV-signal at 300 nm [24]. The current study has described contributions of UA at three lower wavelengths where large variations in contributions may occur, which leads to a need for a multiwavelength approach.

During the dialysis both the contribution of UA and that of PAR metabolites decrease slightly on account of other chromophores. It can be interpreted as the result of increased relative contribution of protein-bound solutes in the dialysate to the total UV absorbance due to quicker removal of water-soluble fraction, but changes are quite small and with remarkable deviations. The same concerns changes followed at 254 nm, which scarcely can be altered in this study by PAR and metabolites with absorbance maximum is in this region [25]. Those results by our group confirm that also the total UV-absorption of the spent dialysate as a marker for dialysis adequacy assessment marks quite closely the same range of small water-soluble uremic solute removal as urea analysis in the serum of patients and the UA analysis in the spent dialysate. On the other hand, it means that UV-monitoring has also the same deficiencies as the urea analysis and cannot add a substantially new quality to the adequacy assessment in addition to immediacy and handiness already verified. 

Since the removal of protein bound solutes is a highly relevant topic in the current dialysis practice [26], an alternative approach was proposed in this work by grouping the peaks-solutes also according to the removal ratios (Table 3). Surprisingly, it turned out that the RR-values of the prevalent majority of UV chromophores are quite comparable with those of the small uremic toxin UA and only the protein bound uremic toxin IS is the single clearly distinguishable UV peak on the chromatograms, which can be used as a marker of a slowly removable fraction for online monitoring of the dialysis process. Unfortunately, the total contribution of IS to the total absorbance is so negligible, from 0.3% at 254 nm up to 2.5% at 280 nm (Figure 4), that it seems impossible to follow the removal of this marker and protein bound solutes in total by means of online UV monitoring of the dialysis. A novel promising method has been proposed lately for monitoring the removal of IS, a known protein-bound solute and uremic toxin, with a low removal ratio, utilizing fluorescence [27].

## 4. Materials and Methods

### 4.1. Clinical Study

The study was performed after approval of the protocol by the Regional Ethical Review Board, Linköping, Sweden. A written informed consent was obtained from all participating patients. The study included eight patients, one female and seven male, mean age 77 ± 7 years, being on chronic three-weekly hemodialysis (HD) and high volume post-dilutional online-HDF (ol-HDF) treatment at the Department of Nephrology, Linköping, Sweden. A high-flux dialyzer FX 80 during the HD sessions and FX 800 during ol-HDF was used and the dialysis machine was Fresenius 5008H (all from the Fresenius Medical Care, Germany). The dialysate flow was 500 mL/min, the blood flow varied between 280 and 350 mL/min. The auto subsystem mode for the calculation of the online prepared substitution fluid by the dialysis machine was used, based on total protein and hematocrit. The substitution fluid volume during the ol-HDF sessions varied between 12.2 and 29.7 liters per session (mean 21.9).

Patient treatments were monitored during three consecutive hemodialysis sessions with duration from 180 to 270 min (totally 24 HD and 24 ol-HDF sessions). During the dialysis the following dialysate samples were taken: (1) 10 min after the start of the dialysis session; (2) at the very end of the treatment, and (3) from the dialysate/ultrafiltrate collection tank after careful stirring. Sampling at the moments of self-tests of the dialysis machine was avoided. Pure dialysate was collected as the reference solution before the start of a dialysis session, when the dialysis machine was prepared for startup and the conductivity was stable. 

### 4.2. HPLC Study

All dialysate samples were acidified down to pH 4.0 with formic acid before the high performance liquid chromatography (HPLC) analysis for conformation with the pH of the chromatographic eluent used. The HPLC system consisted of a quaternary gradient pump unit, a column oven, and a diode array spectrophotometric detector (DAD, all Ultimate 3000 Series instruments from Dionex (Sunnyvale, CA, USA), and Zorbax C18 4.6 × 250 mm column from Agilent Instruments (Wilmington, DE, USA) with a security guard KJO-4282 from Phenomenex (Torrance, CA, USA). The eluent was mixed with 0.05 M formic acid adjusted to pH 4.0 with ammonium hydroxide (A), HPLC grade methanol (B) and HPLC-S grade acetonitrile (C), both from Rathburn (Walkerburn, Scotland). The three-step linear gradient elution program was used, as specified in Table 5.

**Table 5:** HPLC gradient program.

Step	Time (min)	Buffer (A) %	Methanol (B) %	Acetonitrile (C) %
0	0	100	0	0
1	30	60	36	4
2	5	10	81	9
3	4	10	81	9

The total flow rate of 1 mL/min was used continuously at the column temperature of 30 °C. The UV absorbance was monitored at 210, 254 and 280 nm with a measurement interval of 500 ms. Spectra were registered between 200 and 400 nm with a time interval of 0.50 s, data was processed by Chromeleon 6.8 software (Dionex, USA).

Every peak in the HPLC chromatograms was characterized by the characteristic absorption spectrum and by the retention time. Peaks were identified by comparing the retention time, absorption spectrum and MS/MS mass spectrum data (micrOTOF-Q II, Bruker, Germany) of a compound found in the sample with a pure authentic compound. The relative contribution (RC) for the i-th chromatographic peak (presumably solute) to the sum of UV absorbances of all peaks for a chromatogram was calculated as a ratio of the area of the i-th peak (A_peak i_) to the total area of all peaks appeared on the chromatogram (A_total_):


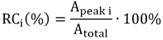
(1)

The contribution values of peaks with similar retention time were averaged separately and depending on the sampling time for all spent dialysate samples from the start and end, also for all of the samples in total. Relative contribution (*RC*) for a specific solute group “j” was calculated analogically to *RC_i_*:


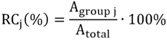
(2)

The removal ratio (RR) of a specific *i*-th peak (uremic solute) for a dialysis session was defined as a function of the start and end HPLC peak areas (A_start *i*_and A_end *i*_) of the samples from the dialysis session:


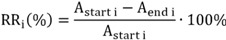
(3)

Statistical analysis was done with Microsoft Excel 2010 (Microsoft Corporation, USA). Student’s *t*-test was used to compare Two-Sample dataset, Assuming Unequal Variances, while *p* < 0.05 was considered significant.

## 5. Conclusion

The focus of this study was on the contributions of the different chromophores in the UV-absorbance signal of the spent dialysate at different wavelengths. UV signal has been proven to describe elimination of easily dialyzed uremic solutes with a high removal ratio [13,14], fully confirmed by the results published in this article. A predominant part of the UV absorption comes from uremic solutes with a high removal ratio not depending on the wavelength of measurements (Figure 4); among these the small molecule of the uric acid is of major importance. The contribution values have high standard deviation values, which indicate remarkable differences of contributions between different dialysis sessions and different patients. At the same time, significant appearance of Paracetamol and its metabolites was detected in the UV-signal, showing that not all of the major UV-absorbing solutes are uremic toxins. While the UV absorbance signal describes the removal of uremic solutes with a high removal ratio very well, it provides scarce information about other molecules, such as slowly removed uremic solutes like indoxyl sulfate. Therefore, the search for a universal, trustworthy, robust and cheap non-invasive dialysis monitoring method is still ongoing.
